# Biologically Inspired Smart Release System Based on 3D Bioprinted Perfused Scaffold for Vascularized Tissue Regeneration

**DOI:** 10.1002/advs.201600058

**Published:** 2016-04-15

**Authors:** Haitao Cui, Wei Zhu, Benjamin Holmes, Lijie Grace Zhang

**Affiliations:** ^1^ Department of Mechanical and Aerospace EngineeringDepartment of MedicineDepartment of Biomedical EngineeringThe George Washington University3590 Science and Engineering Hall 800 22nd Street NWWashington DC20052USA

**Keywords:** 3D bioprinting, biologically inspired smart release, nanocoating, spatiotemporal coordination, vascularized bone regeneration

## Abstract

A critical challenge to the development of large‐scale artificial tissue grafts for defect reconstruction is vascularization of the tissue construct. As an emerging tissue/organ manufacturing technique, 3D bioprinting offers great precision in controlling the internal architecture of a scaffold with preferable mechanical strength and printing complicated microstructures comparable to native tissue. However, current bioprinting techniques still exhibit difficulty in achieving biomimetic nano resolution and cooperating with bioactive spatiotemporal signals. In this study, a comprehensive design of engineered vascularized bone construct is presented for the first time by integrating biomimetic 3D bioprinted fluid perfused microstructure with biologically inspired smart release nanocoating, which is regarded as an aspiring concept combining engineering, biological, and material science. In this biologically inspired design, angiogenesis and osteogenesis are successively induced through a matrix metalloprotease 2 regulative mechanism by delivering dual growth factors with sequential release in spatiotemporal coordination. Availability of this system is evaluated in dynamic culture condition, which is similar to fluid surrounding in vivo, as an alternative animal model study. Results, particularly from co‐cultured dynamically samples demonstrate excellent bioactivity and vascularized bone forming potential of nanocoating modified 3D bioprinted scaffolds for human bone marrow mesenchymal stem cells and human umbilical vein endothelial cells.

## 1 Introduction

Vascularization of large‐scale artificial bone tissue grafts is the most critical challenge for various large bone defect reconstruction. Traditional tissue engineering has been focusing on combining osteoconductive scaffolds, osteoinductive growth factors, and osteogenic precursor cells to repair and regenerate bone. However, nutrient and waste exchange between individual cells and capillary vessels in bone is limited to distances of 100–300 μm. Therefore, construction of vascularized bone grafts plays a vital role in regenerating and remodeling bone tissue.^1^ The blood vessels in native bone are critical for transport of oxygen and nutrients to maintain skeletal tissue functions. Failed vascularization in implanted grafts results in necrosis of osteoblast in the interior and poor integration between neo and host tissues.^2^ So far an ideal vascularized bone construct has not been produced, despite a great deal of research and effort. The main reasons are the deficiencies of perfused vascular structure in hierarchical bone scaffold design, and the effectively targeted stimulation of multiple functional signals.

Currently, the strongly desired characteristics of advanced tissue scaffolds in the field involve both biomimetic properties in structure and the ability to regulate the cell behavior. Hence, an ideal vascularized bone scaffold that can integrate structure with functionality should be designed to regulate osteogenesis and angiogenesis. The engineering techniques that mimic the critical aspects of natural healing and growth cascade, is widely utilized to artificially augment the proliferation and differentiation of the recruited or implanted cells via the integration of growth factors and cytokines that providing suitable biochemical and physicochemical factors for tissue regeneration. Therefore, combining the design of a 3D biomimetic fluid perfused scaffold and an effective growth factor delivery method is regarded as a highly promising technique for vascularized bone regeneration research, especially for eventual clinical applications.^3^, ^4^, ^5^

Regarding 3D scaffold fabrication techniques, phase separation, freeze drying, porogen leaching, and electrospinning may offer limited control over scaffold geometry, pore characteristics and internal channel architecture. All the deficiencies significantly decrease nutrient transportation, cell migration and survival.^6^ Compared with traditional manufacturing technology, 3D bioprinting can provide the ability to construct multiple hierarchical and multi‐scale bone‐like scaffolds with controlled macro shape, porosity and microstructure, thus allowing for patient‐specific fabrication and customized clinical application.^7^, ^8^ 3D bioprinting with fused deposition modeling (FDM) has been one of most effective way to make macro‐scale bone implants with high mechanical strength which also contain microstructures with controllable features. However, the potentially high temperature used to process most common materials for this technique makes it difficult to incorporate bioactive components into scaffolds or include bioactive growth factors delivery.^6^ In addition, current 3D bioprinting techniques (including FDM) exhibit difficulty in achieving biomimetic nano resolution for regulating cellular events.^8^, ^9^ Therefore, the surface modification or other post fabrication technologies are no doubt promising choices to improve biocompatibility and functionality of 3D bioprinted scaffolds.

Within the complex cascade of biological events, growth factors are well known to play a crucial role in regulating cellular behaviors and transferring signals between cells and their extracellular matrix (ECM) to stimulate endogenous repair and regeneration mechanisms, thereby leading to an accelerated functional restoration of damaged or defective tissues. The growth factors that are administered in their native form and without any protection are susceptible to biodegradation and can be rapidly eliminated from the blood circulation, resulting in insufficient amounts at targeted site for a worse therapeutic effect. Although direct adsorption, layer‐by‐layer (LbL) technology, multiphase loading, particulate‐based delivery, hydrogel‐based delivery, and their combination application as well as some intelligent delivery systems have been developed over the past decade, targeted transport and sustained release of growth factors with time‐ and dose‐dependent profiles still have little achievement.^3^, ^10^, ^11^, ^12^ Incorporating smart stimuli‐responsive elements into growth factor delivery system is one highly innovative strategy to obtain specific release triggered by external stimuli. Drug or gene delivery in response to pH, temperature, magnetic, ultrasound, irradiation and electric stimuli has shown great promise, however, the delivery of growth factors via external triggers for tissue engineering remains limited to their intrinsic characteristics, including deactivation by exogenous stimuli due to poor protein stability, and poor encapsulation or release effects due to relatively large size.^11^, ^13^ More importantly, few systems have addressed the cooperative biological signaling events of cells as a function of the changes in their dynamic microenvironment. The state of the art concept toward the delivery of dual or multiple growth factors is not only to make more efforts for developing sophisticated delivery platforms, but to explore a biologically inspired system that dynamically release multiple cues to regenerate complex tissues and more closely reproduce the evolving microenvironment that occurs in natural ECM.

Therefore, with development of tissue engineering technology, new scaffold manufacturing technique and smart growth factor delivery approaches are strongly desired to develop forward to comprehensive engineering design and biologically inspired responsive induction. Consequently to vascularized bone regeneration, current synergistic therapy also lacks a biologically active control mechanism for responsive multiple growth factor delivery to induce angiogenesis and osteogenesis in spatiotemporal coordination.^12^

**Figure 1:**
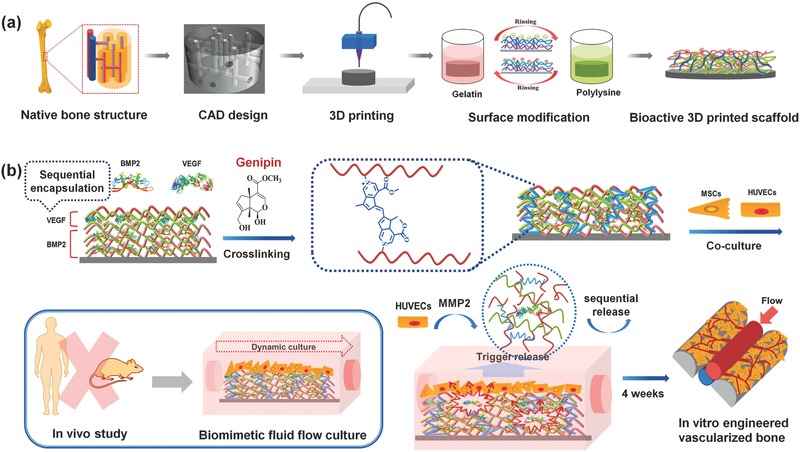
a) Schematic illustration of the fabrication process of nanocoating modified 3D bioprinted scaffolds. According to the native bone structure, the biomimetic perfused scaffold combining bone support and vascular channels was designed and printed by FDM printer. Then surface modification process was performed to obtain a bioactive vascularized bone construct. b) Schematic representation of sequential adsorption and biologically inspired release of growth factors in the nanocoating film. The rhBMP‐2 was adsorbed in first 15 dual‐layers and then rhVEGF was adsorbed in the top 5 dual‐layers together with genipin crosslinking reaction. When MSCs and HUVECs were co‐cultured in dynamic fluid, the secretion of MMP2 by HUVECs could trigger the release of growth factors. After 4 weeks of culture, the vascularized bone structure would be formed in vitro.

Hence, there is a strong requirement for a vascularized bone scaffold that can integrate biomimetic structure with functionality to intelligently regulate osteogenesis and angiogenesis. In this study, we implemented an integrated set of manufacturing processes for the first time which combines biomimetic 3D structure design with post fabrication functionalization (**Figure** **1**a,b). Research activities included: (1) bioprinting a 3D fluid perfused microstructure vascularized bone scaffold via computer‐aided design (CAD) and (2) fabricating a biologically inspired smart release nanocoating on the surface of the bioprinted scaffold to coordinate spatiotemporal angiogenic and osteogenic growth factor delivery. This engineered vascularized bone constructs were cultured in dynamic fluid surrounding which may provide an alternative to sacrificed animal experiment, to evaluate the availability of biologically inspired smart release system for improved vascularized bone regeneration.

## 2 Results

### 2.1 Biomimetic Engineered Complex Tissue Scaffold Bioprinting and Post Fabrication

**Figure 2:**
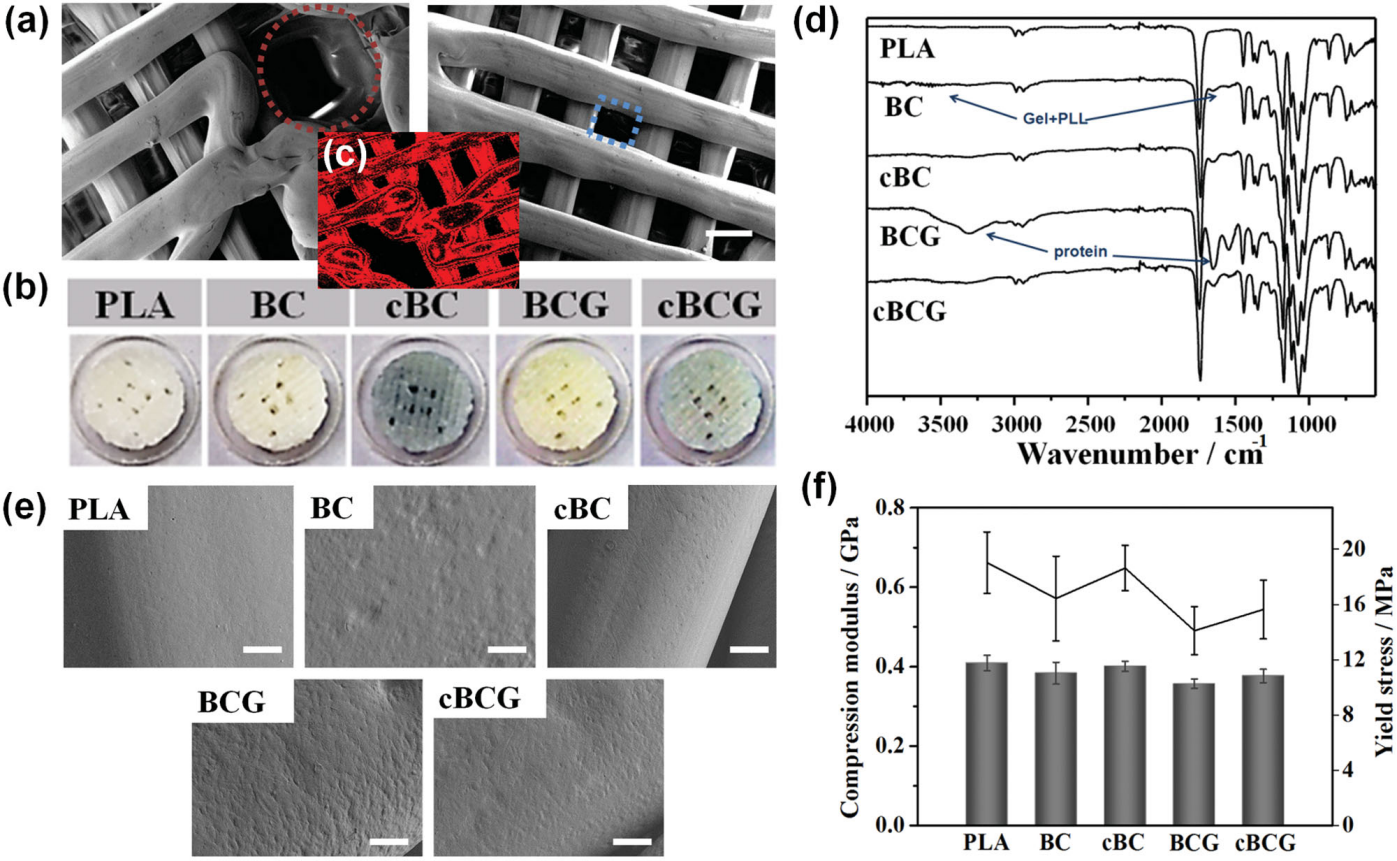
a) Microstructural characterization of 3D bioprinted perfused scaffold based on CAD design by SEM. The red circle shows 500 μm vascular channels and the blue square shows 200 μm pores of bone scaffold. The scale bars indicate 200 μm. b) Images of different scaffolds, including PLA, bioactive nanocoating (Gel/PLL)_20_ modified PLA (BC), Gnp crosslinked bioactive nanocoating [(Gel/PLL)_20_]GnP modified PLA (cBC), bioactive nanocoating with growth factors (BCG), and Gnp crosslinked bioactive nanocoating with growth factors (cBCG). c) Red auto‐fluorescent image of cBC or cBCG. d) ATR‐FTIR spectra of different scaffolds. e) Surface morphologies of the different coating modified scaffolds, untreated PLA served as a control. A nanoscale islet‐like feature uniformly distributed over the surface and the adsorption of protein increased the roughness, whereas the crosslinking process weakened these changes. f) Mechanical properties of 3D bioprinted scaffolds. After the post fabrication modifying process, 3D bioprinted scaffolds maintained native bone‐like mechanical strength.

Through the optimization of the engineering design, we have successfully obtained a 3D bioprinted vascularized bone construct with a unique integration of fully interconnected microvascular network within a microstructured bone matrix. Within this vascularized bone model, “square pore shaped” scaffolds were composed of stacked units with a 200 μm line distance and a 250 μm layer height to form a porous cylinder. In order to mimic the arrangement of blood vessels in native bone, a series of interconnected horizontal and vertical channels (500 μm) were designed as shown in **Figure** **2**a. The microvascular design of the constructs can possess similar flow characteristics to native blood vessels under pulsatile arterial flow as demonstrated in our recent study.^14^ 3D models were printed using polylactic acid (PLA) on a FDM printer. Afterward, a novel and simply implemented surface modification strategy was employed to provide a nanoscale surface feature and immobilize bioactive cues onto the biomimic 3D scaffolds. Gelatin (Gel) and polylysine (PLL) with sequential adsorption of dual growth factors (recombinant human bone morphogenetic protein, rhBMP‐2 and recombinant human vascular endothelial growth factor, rhVEGF), were assembled layer by layer on the 3D scaffold via electrostatic interaction to form (Gel/PLL)_20_ multilayer nanocoatings. The multilayer coating was then crosslinked by genipin (GnP) to form interpenetrating polymer networks (IPN) [(Gel/PLL)_20_]_GnP_. Since human umbilical vein endothelial cells (HUVECs) express matrix metalloprotease 2 (MMP2) which is a type of gelatinase with the capacity to degrade gelatin to short peptide chains.^15^ With the progression of vascular development and subsequent MMP2 accumulation, the crosslinked networks could be cleaved to release the growth factors. Therefore, a comprehensive design of engineered vascularized bone scaffold was presented for the first time which integrated biomimetic 3D printed structures with organic self‐modulatory mechanisms. Compared with traditional growth factor release system, this design can not only inherit all superiorities from LbL adsorption, but also be endowed with a particular desirable ability of biologically inspired release.

### 2.2 Biologically Inspired Smart Release Coating Fabrication and Characterization

The interactions between cells and biomaterials are mainly dependent on the physicochemical characteristics of the biomaterials' surfaces.^16^ It is expected that this nanocoating could improve surface properties and provide a special functional domain for the 3D bioprinted scaffold to promote cell–substrate interaction.^17^ Gel, a negatively charged biopolymer, consists of highly bioactive polypeptides that are derived from collagen. Numerous RGD (arginylglycylaspartic acid) integrins and other functional recognition sequences within gelatin are beneficial for cell attachment, migration, proliferation, and differentiation. Positively charged PLL is widely used to promote cell adhesion via enhancing electrostatic interaction with negatively charged ions of the cell membrane. In this study, the 20 dual‐layer assembly were designed to optimize the contribution of bioactive components and improve the loading of growth factors. The crosslinking process may also further stabilize the multilayer coating and avoid burst release of growth factors. After layer by layer assembly, the remaining amino groups from Gel and PLL contributed to the crosslinking reaction with GnP. GnP as a crosslinking agent in this reaction is an enzymatic product of geniposide isolated from the fruit of the gardenia plant and is reacted with free amino groups to form blue pigments.^18^ Hence, a blue coating was observed on the surface of scaffold after the crosslinking reaction. Moreover, the coated scaffold exhibited a strong red fluorescence due to the intrinsic red fluorescence of GnP, illustrating that it could maintain this unique property for diagnostic imaging (Figure 2b,c). ATR‐IR spectroscopy clearly confirmed the successful preparation of a GnP crosslinked nanocoating and effective loading of growth factors on the surface of 3D bioprinted scaffold (Figure 2d). Plain PLA scaffolds exhibited a hydrophobic surface with an average contact angle of 75°. Through the surface modification, there was a distinct increase in hydrophillicity (contact angle about 50°) for the nanocoating (Figure S1, Supporting Information). Morphology analysis revealed a nanoscale islet‐like feature uniformly distributed over the surface and the adsorption of growth factors increased the surface roughness when compared with smooth and featureless PLA (Figure 2e). The crosslinking process made these features more homogeneous and compact, which further increased interface stiffness. The assembly of the bioactive components not only affected the hydrophilicity of the substrate, but also changed the surface morphology, which would in turn influence the cell behaviors on the scaffolds. After the post fabrication modification, the 3D bioprinted scaffolds maintained excellent mechanical properties (Figure 2f). They possessed a native bone‐like mechanical strength, with a compress modulus of about 0.4 GPa and a yield stress higher than 15 MPa. This could provide a desired support for bone regeneration. Therefore, surface modification may be one of the most direct and effective strategies to improve the biocompatibility of scaffolds and modulate cellular events without causing a significant change to the intrinsic mechanical and microstructure properties of designed synthetic grafts. We also found these bioprinted PLA scaffolds exhibited unobvious degradation behavior in PBS or esterase solution during 4 weeks of culture, thus it can provide a stable surrounding for our nanocoating release system further to promoting tissue regeneration (Figure S2, Supporting Information).

**Figure 3:**
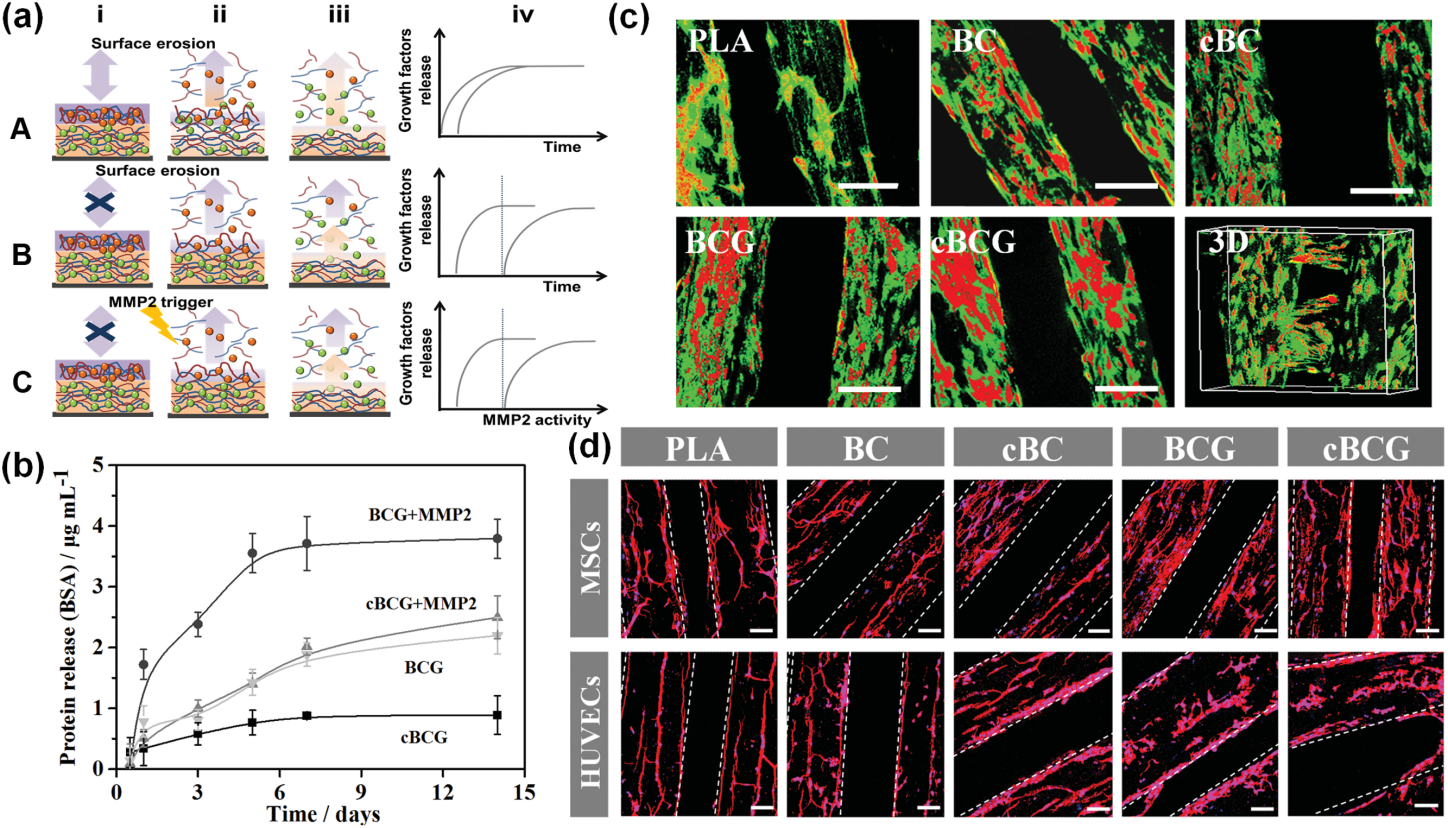
a) Illustration of the proposed assembly (i) and release (ii, iii) process of multilayer films without (A) and with (B) crosslinking, as well as our biologically inspired system (C), where the BMP2 (green spheres) and VEGF (red spheres) are loaded into films composed of PLL (blue) and MMP trigger‐cleavable Gel (red). Compared with traditional LbL film adsorption, crosslinking retain their stable immobilization and sequential release without highly inter‐diffusion. Moreover, surface erosion contributes film degradation where the therapeutic agent is released throughout the film, whereas biologically inspired system exhibits a controllable release behavior. The release profiles reflect the effect of crosslinking, and biologically inspired on kinetics of drug release (iv). b) Protein release profiles of nanocoating with BSA within 2 weeks. The cBCG could sustainably release up to 4 weeks (not shown in here). MMP2 was thought to trigger the cleavage of gelatin chain to controllably release growth factors. c) Confocal fluorescence images of hMSCs and HUVECs co‐culture on various scaffolds in a static culture condition for 5 days. hMSCs were labeled with cell tracker green, and HUVECs were stained with cell tracker red, respectively. The scale bars indicate 200 μm. The cBCG scaffold was also imaged as 3D scanning structure. d) Fluorescent images of hMSCs and HUVECs on the 3D bioprinted scaffolds with F‐actin (red) and nucleus (blue) staining in a static culture condition for 3 days. The hMSCs exhibited a well distributed spread on scaffold surface, while the HUVECs formed an aggregative microvascular networks. The scale bars indicate 100 μm.

rhBMP‐2 is an osteogenic growth factor used extensively in both ectopic and orthotopic sites for bone generation. rhVEGF is an angiogenic factor critical for both intramembranous and endochondral bone formation. Dual application of rhBMP‐2 and rhVEGF has been regarded as one of the most efficient system for effective vascularized bone formation.^5^, ^19^, ^20^ However, traditional delivery techniques have exhibited an unfavorable therapeutic effect. A burst release and low sustained doses of growth factors have a limited effect for the long term bone regeneration, while an excess of rhBMP‐2 may lead to undesirable incidences of hematoma, ectopic bone formation, and osteoclast induced osteolysis.^21^ Additionally, excess amounts of rhVEGF can actually inhibit osteogenesis, associating with severe vascular leakage and hypotension.^22^ Therefore, the amount and timing of rhBMP‐2 and rhVEGF delivery is critical to enhance bone formation and localized vascularization simultaneously.^20^, ^23^ The smart nanocoating used in our design can not only be utilized to immobilize bioactive components onto biomaterial surfaces, but also to control the growth factors quantity and sequential release. More importantly, our system could control the release of growth factors to regulate cell behaviors through organic self‐modulatory mechanisms during vascularized bone formation. Wherein, the nanocoating was fabricated according to the protocol, with rhBMP‐2 being adsorbed in the first 15 dual‐layers and then rhVEGF layers being adsorbed in the top 5 dual‐layers.^24^ We anticipated that rhVEGF would be initially released from the top layers to stimulate the formation of blood vessels, followed by rhBMP‐2 release for initiating osteogenic differentiation. The crosslinking process could further stabilize the growth factors in the nanocoating and prevent their rapid clearance. **Figure** **3**a illustrates organic self‐modulatory mechanism in our system, which is characteristic of sequential release from crosslinked multilayer films with a representation of the proposed film architecture by biologically inspired manner instead of the simple surface erosion. When using BSA as a model protein and MMP2 as a cleaved trigger to study the release profile (Figure 3b), we found the protein could be sustained released from nanocoating at a minimal dose level over a prolonged time period of several weeks. The crosslinking process also greatly improved the loading stability of BSA in the nanocoating. Moreover, MMP2 could sensitively trigger the fracture of IPN to release protein. The goal being the creation of an effective release mechanism performed successfully on the cBCG scaffold. Instead of uncontrollable diffusion process by surface erosion in traditional LbL system, the controlled nature of localized release from our 3D scaffold surfaces can eventually enables much lower doses of growth factors to be effective for tissue regeneration.

### 2.3 Human Mesenchymal Stem Cells (hMSCs) and HUVECs Co‐Culture on 3D Bioprinted Scaffold

Some studies have indicated a positive effect of implanting biomaterial constructs co‐cultured with mesenchymal and vascular cells, where the development of vascularized tissues both in vitro and in vivo was enabled.^25^ Therefore, co‐culturing hMSCs with HUVECs in our study was conducted to generate the vascularized bone tissue. The cellular organization of co‐culturing hMSCs and HUVECs on the scaffolds in a static culture condition was investigated after 5 days. Images of green labeled hMSCs and red labeled HUVECs showed that hMSCs homogeneously distributed on the surface of scaffolds. Meanwhile, HUVECs were inclined to aggregate and migrate to form line patterns on the scaffolds (Figure 3c). In addition, both hMSCs and HUVECs on the nanocoating modified 3D bioprinted scaffolds exhibited excellent adhesion and proliferation, compared with an unmodified PLA control (Figures S3 and S4, Supporting Information). F‐actin staining showed that on the nanocoating, hMSCs spread well and maintained a spindle morphology, whereas HUVECs preferred to grow in lines and form highly aligned network structures (Figure 3d).

### 2.4 *In Vitro* Engineered Vascularized Bone Construction on Dynamic Culture Condition

**Figure 4:**
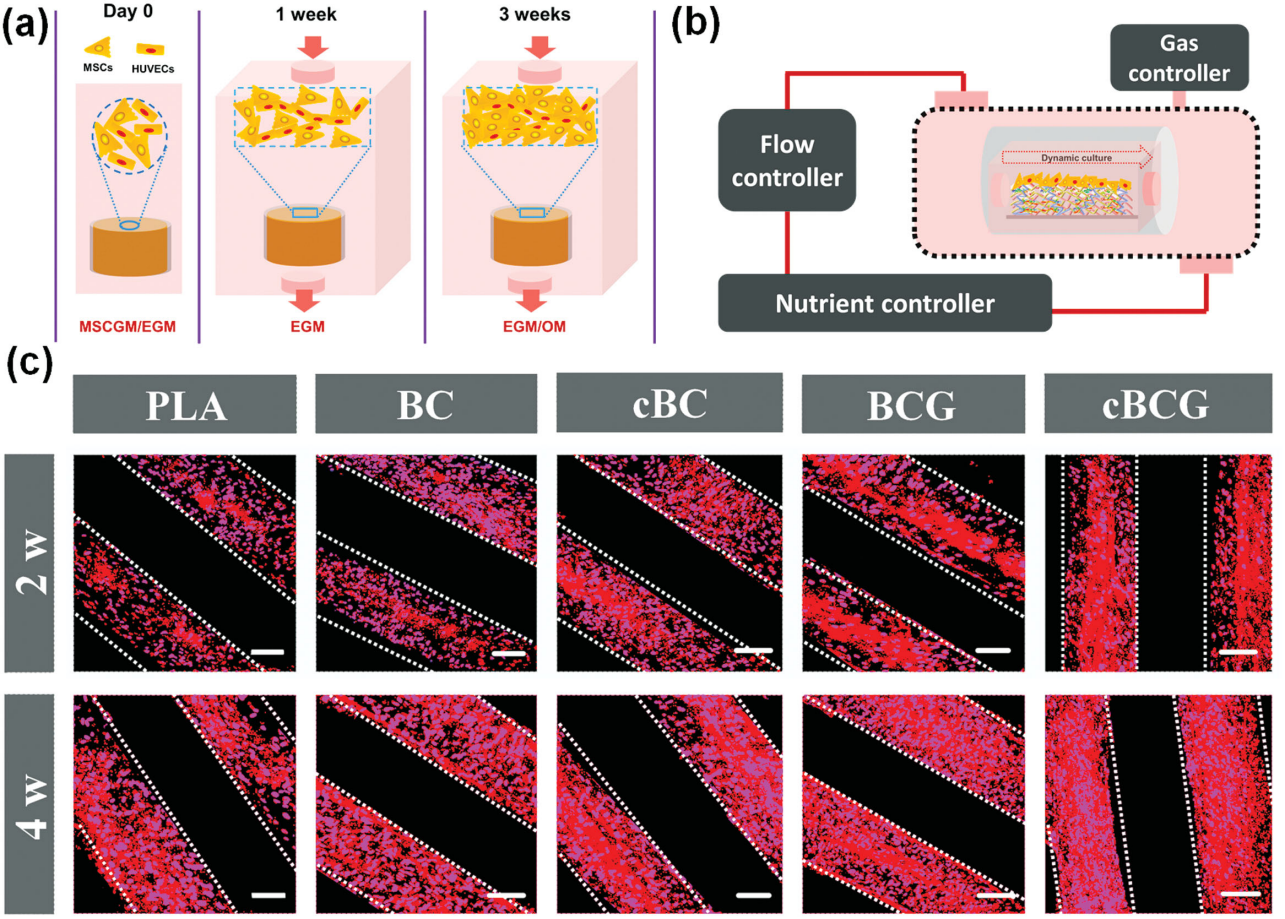
a) Schematic illustration of experimental approaches. hMSCs and HUVECs were seeded in EGM and MSCGM at 1:1 ratio on scaffolds for first day. Then the vascular differentiation was induced for 1 week in EGM. At last, the OM/EGM (1:1) was supplied to induce osteogenic differentiation for another 3 weeks. b) Schematic diagram of dynamic culture in a custom‐designed flow bioreactor system. The system composes of four parts, which are perfused chamber, flow controller, nutrient controller, and gas controller. When culture medium flowed through constructs, the cells seeded on the scaffolds would be subject to fluid shear stress by mimicking fluid surrounding in vivo. c) Immunofluorescence staining of the vascularization marked with CD31 antibody for 2 and 4 weeks in a dynamic culture condition. The scale bars indicate 100 μm.

In order to generate a functional vasculature prior to osteogenic induction, we developed a two‐step culture protocol (**Figure** **4**a). hMSCs and HUVECs were co‐cultured in endothelial growth media (EGM) for a week to induce the formation of vascular networks, and then incubated in osteoinductive media (OM)/EGM (1:1) for 3 weeks to induce bone formation. Moreover, to mimic the unique flow characteristics of the native vascularized bone microenvironment, a dynamic culture was conducted to investigate vascularized bone formation (Figure 4b). The biomimetic‐engineered strategy was adopted in our customized flow fluid device as an alternative method of animal studies. Such conditions, when combined with our highly perfused scaffold, are beneficial to the formation of microvascular structures.^26^ The immunofluorescence images of MSCs and HUVECs co‐culture showed faster and higher CD31 expression on our cBCG scaffold within 4 weeks induction, suggesting an ongoing process of perivascular coverage of capillaries induced from sustainable VEGF release (Figure 4c and Figure S5, Supporting Information). On one hand, the fluid shear stress was performed on our perfused scaffold to accelerate microvascular formation through mimicking fluid surrounding in vivo; on the other hand, sustainable release of VEGF further promoted partial MSCs endothelialization and angiogenesis.

**Figure 5:**
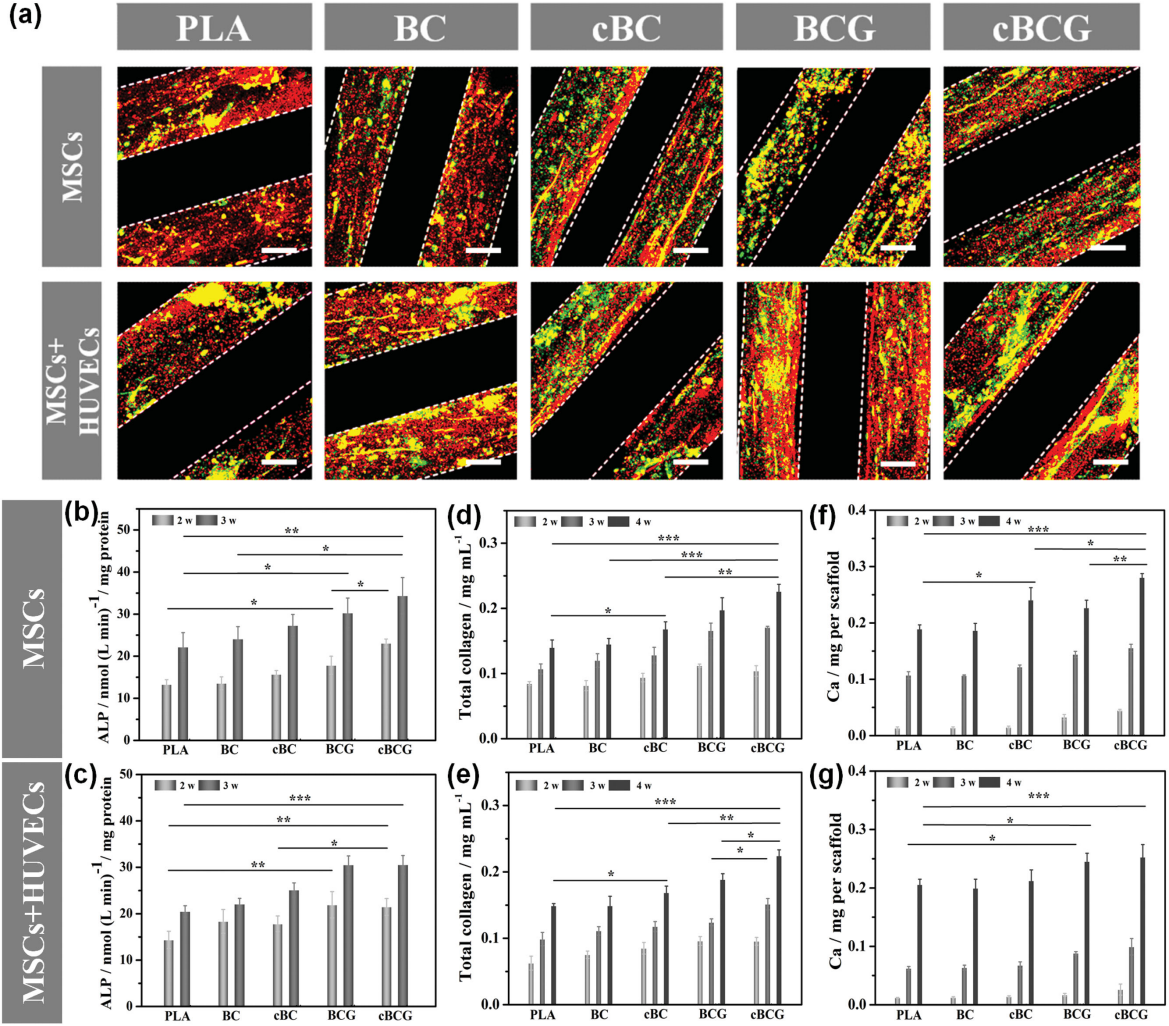
a) Immunofluorescence staining of the vascularized bone formation in the dynamic co‐culture condition. The fluorescence images for anti‐vWF (green) and OPN (red) showed that the cBCG scaffold possessed more vascular‐like network and osteogenesis than other control groups. The scale bars indicate 100 μm. b,c) Quantification of ALP activity. d,e) Total collagen synthesis. f,g) Quantification of calcium deposition content on different scaffolds comparing the dynamic co‐culture with dynamic monoculture. All data showed that the cBCG scaffold enhanced the osteogenic differentiation.

To verify the self‐modulatory release ability of our scaffolds in the presence of HUVECs, a monoculture of hMSCs was conducted as a control in vascularized bone differentiation study. After 4 weeks of culture, the maturation of bone and vascular tissue on the scaffolds was assessed using immunofluorescence staining of the osteogenic differentiation marker osteopontin (OPN, red) and angiogenic specific marker von Willebrand factor (vWf, green), respectively (**Figure** **5**a). In previous studies, hMSCs have been reported to differentiate into endothelial cells in the presence of rhVEGF, and hMSCs possessed the potential to directly form vascularized bone.^27^ The hMSC monoculture displayed some evidence of vascular formation, however, the HUVECs inducted from MSCs showed a limited positive effect on the growth factor release triggered by the MMP2. In contrast, a marked maturation on vascularized bone was observed in the co‐culture system. This was a reasonable and expected result since a high density of endothelial cells in the co‐culture system shortened vascularization time.

As discussed, sequential adsorption allowed for rhVEGF release firstly from the nanocoating for inducing vascular formation. Then rhBMP‐2 was released to upregulate osteogenic differentiation. The nanocoating scaffold adsorbed with duel growth factors exhibited a higher expression for specific differentiation markers relative to other control groups. It is postulated that the crosslinked nanocoating modified scaffold released growth factors through a MMP2 regulative mechanism instead of diffusion effect. MMP2 secreted by HUVECs would act as on–off switch for the growth factor release, as the activation of the release system depends on the MMP2 expression to cleave the IPN. The nanocoating modified scaffolds with hMSCs and HUVECs co‐culture not only possessed excellent bone forming potential, but also exhibited well‐developed and aggregative microvascular networks. As a structure's innovative design of 3D bioprinted scaffolds, the microchannel networks present in our scaffolds are beneficial to the integration of neovascular formations into native vasculature in the implantation site. This would enable the formation of a circular and stable network, which is a preceding step to creating mature blood vessels in engineered new bone.

hMSC osteogenic differentiation on various scaffolds was evaluated quantitatively by measuring alkaline phosphatase (ALP) activity (an early osteogenic differentiation marker), determining total collagen expression (which is main component for bone ECM), staining for bone mineralization, and quantifying calcium content. A rapid increase and high expression in the ALP activity in a short period was observed in all growth factor loading groups (Figure 5b,c). In our design, the number of hMSCs in co‐culture group was one half of that in monoculture group. However, the two groups exhibited similar results on osteogenic differentiation. Compared to the hMSC monoculture, the ALP activity of hMSCs on the cBCG scaffold in the co‐culture system which triggered‐release the rhBMP‐2 with sustained low dose modality by HUVECs exhibited more rapidly increase with prolonged expression. Due to the initial burst release, the ALP activity on the non‐crosslinking nanocoating did not show any significantly sustained improvement. Therefore, the rhBMP‐2 could be well stabilized in the crosslinked networks and efficiently controlled release achieved with a prolonged time in the co‐culture system. The synthesis of total collagen was also evaluated to verify these characteristics and effects (Figure 5d,e). Compared with the control groups, hMSCs on the BCG and cBCG scaffolds expressed significantly higher collagen by rhBMP‐2 release. In addition, the collagen content of hMSCs on the cBCG scaffold in the co‐culture system was significantly higher than that of non‐crosslinking group or monoculture groups. For the longer differentiation period, this controlled release behavior was desired to produce beneficial effect over the duration of the experiment, avoiding a rapid clearance of growth factor.

Mineralization is ultimately the most important indicator of hMSC osteogenic differentiation, thus the calcium deposition on all scaffolds was investigated after 4 weeks of culture (Figure S6, Supporting Information). Compared with bare PLA scaffold, all nanocoating modified scaffolds showed a positive effect of mineralization. These results could be attributed to the charged surface which serves as a binding site for calcium ions or acidic phospholipids and as nucleation sites for mineralization. In addition, the crosslinked nanocoating (cBC and cBCG) presented an improved calcium deposition when compared with the non‐crosslinked nanocoating, suggesting that the increased surface stiffness could be beneficial to overall mineralization. This phenomenon was also observed in other papers, the matrix stiffness at the cell‐implant interface resulted in the greatest enhancement of the osteogenic differentiation.^28^ A larger area of continuous Alizarin red staining was observed in those groups adsorbed with rhBMP‐2. In particular, the intensity of the staining and the size of the deposit were greatest on the cBCG scaffold in the co‐culture system. Similar to the staining results, calcium content analysis further confirmed those phenomena (Figure 5f,g). The differentiation results demonstrated that the cBCG scaffold could provide a biomimetic bone‐like structure and regulate the release of growth factors for extended time periods to promote vascularized bone formation. We also studied the osteogenic differentiation of hMSC on the scaffolds in static culture conditions (Figure S7, Supporting Information). Compared with BCG scaffolds, our cBCG scaffolds provided stable performance in the dynamic fluid environment similar to in the static culture. Therefore all results indicated that, via the crosslinking process, the cBCG scaffold would theoretically have excellent properties on for efficient and enhanced, yet regulated vascularized bone formation in vivo.

## 3 Discussions

For engineered tissue regeneration, the hierarchical and complicated tissue structure is difficult to precisely fabricate through traditional manufacturing technique of scaffolds. Although 3D bioprinting as an advanced manufacturing technology can precisely fabricate the internal macro‐architecture and complicated microstructures of scaffolds, current bioprinting techniques are still difficult to obtain nanoscale feature and directly cooperating with bioactive signals with controllable manner. Except for advanced structural design, determining the roles that growth factors play in tissue repair and regeneration is as important as designing, developing and applying suitable formulations that release them with spatiotemporal control. As previously reported, LbL assembly provided a simple and effective strategy to modify and functionalize scaffolds. Additionally, the sequential adsorption of multiple growth factors could exhibit release successively to promote tissue regeneration with time dependent kinetics. However, highly inter‐diffusion of polyelectrolyte layers resulted in undesirable leakage of growth factors without sequential release, and driving force of surface erosion made growth factors passively release with negative effects. In view of addressing all this drawbacks, we proposed a state of the art stimuli release manner, “biologically inspired release profile”, which depended on the coordinated interactions with cells or a local cellular microenvironment for triggering changes of delivery systems and thereby leading to controlled release of growth factors.

In this study, we demonstrated that integrating a biologically inspired smart release nanocoating strategy with biomimetic 3D bioprinted fluid perfused microstructure could create a highly innovative vascularized bone construct with nano to micro features and self‐modulatory angiogenic and osteogenic growth factor delivery. In virtue of the precise microstructure of scaffold by 3D bioprinting, this bioactive nanocoating might perform a targeted immobilization of growth factor via proposed assembly protocol. Moreover, biologically inspired release system addressed the cooperative biological signaling events of cells as a function of the changes in their dynamic microenvironment. In this biologically inspired design, angiogenesis and osteogenesis were successively induced through a MMP2 regulative mechanism by delivering dual growth factors with sequential release in spatiotemporal coordination. Therein, crosslinking process greatly improved the loading stability of growth factors in the nanocoating without inter‐diffusion. Availability of this system was evaluated in dynamic culture condition, which was similar to fluid surrounding in vivo, as an alternative animal model study. When culture medium flowed through constructs, the cell seeded on the scaffolds would be subject to fluid shear stress by mimicking fluid surrounding in vivo. Our results demonstrated good bioactivity and vascularized bone forming potential of nanocoating modified 3D bioprinted scaffolds. The ability of such a strategy to intelligently regulate rhBMP‐2/rhVEGF release has great potential for improving vascularized bone regeneration and avoiding undesired harmful side effects in clinical applications.

## 4 Conclusion

Although various 3D fabricated scaffolds, surface modification methods and growth factor delivery strategies have been investigated in biomedical application, integrating engineered perfused design of scaffolds and biologically inspired release system is yet to be explored in the manner of biomimetic hierarchical architecture and dynamic biological signaling events. This study makes use of a modular approach to generate bioactive nanocoating on perfused 3D bioprinted scaffold that release growth factors through MMP regulative mechanism, and demonstrates their stimuli‐responsive profiles toward improving vascularized bone regeneration. These results present a highly innovative release mechanism for growth factor delivery by biologically inspired process, which may not only benefit vascularized bone regeneration, but also extend to improving any complex vascularized tissue or organ regenerations.

## 5 Experimental Section

*Biomimetic Scaffold Design and 3D Bioprinting*: The biomimetic scaffold was designed and printed based on previously reported method. Within this vascularized bone model, the “square pore shaped” scaffolds were composed of stacked units with a 200 μm line distance and a 250 μm layer height to form a porous cylinder. In order to mimic the arrangement of blood vessels in native bone, a series of interconnected horizontal and vertical channels were designed as shown in Figure 1. The diameter of vascular channels was nearly 2.5 times greater than the pore size in the bone regions of the scaffold. The vascular tubes were long interconnected channels, while the pores of bone region were closely arrayed layer by layer to form regular networks. 3D models were printed into scaffolds layer by layer from PLA on an FDM printer. Additionally, representative CAD models of the scaffolds were used to analyze for surface area, volume, and pore density. The theoretical parameters of scaffold structure were calculated, including the wall thickness (≈200 μm), pore size (≈200 μm), porosity (≈50%), channel size (≈500 μm), and surface area/volume ratio (≈30). The 200 μm is regarded as an ideal pore size for the bone scaffolds and the larger channel may provide a biomimetic fluid environment and vascular invasion spaces in vivo.

*Crosslinked LbL Assembly Film Construction*: For the construction of bioactive nanocoating modified 3D scaffold, the biocomponents were fabricated onto 3D bioprinted scaffold surfaces via electrostatic assembly. Briefly, aminolyzed PLA scaffolds were obtained by immersion in PEI solution (5.0 mg mL^−1^) for 12 h. Then, polyanion (gelatin, Gel) solution and polycation (polylysine, PLL) solution (2.0 mg mL^−1^) were alternatively assembled onto the scaffolds via 30 min immersions each, followed by three rinses with PBS buffer, until the desired (Gel/PLL)_20_ architectures were obtained. During the assembly process, rhBMP‐2 and rhVEGF (0.5 mg mL^−1^) were adsorbed into the coatings. The rhBMP‐2 was adsorbed in the first 15 dual‐layers and then the rhVEGF was adsorbed in the top 5 dual‐layers. For the preparation of the IPN, GnP in PBS (0.50%, w/v) was used to crosslink the amino groups of polyelectrolytes. The LbL‐coated scaffold was immersed into GnP solution for 48 h at room temperature, and finally rinsed with PBS. ATR‐FTIR spectroscopy measurements were performed with a Perkin Elmer Spectrum BX system, to detect nanocoating structural changes. The degradation behavior of all scaffolds was studied in PBS and esterase solution for 4 weeks.

*3D Scaffold Mechanical and Morphological Characterization*: The mechanical properties of all scaffolds were tested using MTS criterion universal testing system equipped with a 50 k N load cell (MTS Corporation, US), according to international organization for standardization (ISO) and American society for testing and materials (ASTM). The scaffolds were compressed at a strain rate of 2 mm min^−1^ to a maximum strain of 20%. The slope of the linear elastic region of stress–strain curve was calculated to obtain the compressive modulus. The compressive strength was obtained corresponding to the stress value at the yield point. The morphology and surface topography of scaffolds were studied using a Zeiss SigmaVP scanning electron microscope (SEM). All scaffolds were coated with a roughly 10 nm thick gold layer and imaged using 5 kV electron beam.

*MMP Triggered Controllable Release*: Release studies of nanocoating modified 3D scaffolds were performed using bovine serum albumin (BSA) as protein model by incubation in PBS (pH 7.4) at 37 °C. Relative quantification of protein released from the nanocoating was determined using micro BCA protein assay kit (Thermo scientific). The BSA (1.0 mg mL^−1^) was absorbed into the LbL coating in the assembly process, and matrix metalloproteinase 2 (MMP‐2, 50 ng μL^−1^) was used to cleave the crosslinked nanocoating in the release study. The release media was withdrawn at fixed time intervals and replaced with fresh buffer. The sample solutions were monitored using UV–vis spectrophotometry at 562 nm to determine BSA concentration. The calibration curve was plotted using standard protein solutions with known concentrations of proteins.

*hMSCs and HUVECs Co‐Culture*: hMSCs (Texas A&M Health Science Center, Institute for Regenerative) were cultured in mesenchymal stem cell growth media (MSCGM) consisting of alpha minimum essential media, 20% fetal bovine serum (FBS), 1% l‐glutamine, 1% penicillin/streptomycin. HUVECs (Life Technologies) were cultured in EGM consisting of Medium 200 and low serum growth supplement (LSGS). For osteogenic differentiation studies, hMSCs were cultured in OM (MSCGM supplemented with 10 × 10^−9^ m dexamethasone, 50 μg mL^−1^ l‐ascorbate acid and 10 × 10^−3^ m β‐glycerophosphate (Sigma)). All experiments were performed with hMSCs and HUVECs of six cell passages or less. According to the previous study, a 1:1 ratio was optimally chosen in co‐culture studies as it provided robust and stable vascular networks while enabling bone formation. hMSCs and HUVECs (2 × 10^5^ cells mL^−1^) were incubated with CMFDA and CMTMR (10 × 10^−6^ m Molecular Probes, CellTracker Dye, life technologies) for 30 min at 37 °C, respectively. The cells were mixed in a 1:1 ratio and then cultured on the scaffolds in a static condition for 5 d. The cell location or arrangement on the 3D bioprinted scaffolds in co‐culture system was imaged with a Zeiss 710 laser scanning confocal microscope.

*Cell Adhesion and Proliferation*: To study the effect of nanocoatings on hMSC and HUVEC attachment, the cells (2 × 10^5^ cell mL^−1^) were seeded on various scaffolds for 4 h. The samples were assessed by the 3‐(4, 5‐dimethylthiazol‐2‐yl)‐2, 5‐diphenyltetrazolium bromide (MTT) assay. Briefly, MTT solution (0.5 mg mL^−1^) was added in the plate and then incubated for 4 h. After the media was removed, isopropanol/HCl solution (1 m) was added to dissolve the formazan crystals. The optical density (OD) was measured at 490 nm by photometric plate reader (Thermo Scientific). The cell proliferation was conducted 1, 3, and 5 d. Samples were seeded with 1 × 10^5^ cell mL^−1^ and counted at each time point using the same MTT assay described above. To investigate the effect of surface features on the hMSC and HUVEC phenotype and spreading, the organization of actin filaments of adherent cells cultured on our constructs was evaluated after 3 d culture in the static condition. The cells' cytoskeleton was identified with double staining of actin staining (red) using Texas Red‐phalloidin and nuclei staining (blue) using 4,6‐diamidino‐2‐phenylindole dihydrochloride (DAPI) (Invitrogen). Cells were fixed in 10% formalin for 15 min, permeabilized in 0.1% Triton X‐100, and blocked with 1% BSA. Cells were then incubated with phalloidin for 20 min and DAPI for 3 min. Samples were observed and imaged using a Zeiss 710 confocal microscope.

*In Vitro Vascularized Bone Grafts on Dynamic Culture Condition*: To induce vascularized bone formation, hMSCs and HUVECs (5 × 10^5^ cell mL^−1^) was seeded onto scaffolds, and divided into three culture condition groups including static co‐culture, dynamic co‐culture, and dynamic hMSCs monoculture. A flow bioreactor system was utilized for incubating cells on 3D bioprinted scaffolds to study vascularized bone formation in a dynamic culture. The system consisted of a digital peristaltic pump (Masterflex, Cole‐Parmer), a fluid reservoir with culture medium, and a port for gas exchange with 5% CO_2_/95% air. Efficient transfer of nutrients and oxygen is facilitated by the convective forces provided by unidirectional creep flow through the scaffolds. The optimal culture condition is to utilize EGM for 1 w and then a mixed media composed by EGM and OM at 1:1 ratio for 3 weeks. At predesigned time points, cells were digested in lysed buffer via freezing at −80 °C and thawing at 37 °C. The lysate was collected to test ALP activity and collagen secretion. The ALP activity was determined for 7 and 14 d using ALP assay kit (Bioassay Systems) after the initiation of MSC osteogenic differentiation. ALP substrate was added to the digested suspension in the dark for 30 min, and then the absorbance was read at 405 nm. Measurements were compared to *p*‐nitrophenol standards and normalized to total cell protein. The total collagen content was measured via Sirius red method. The suspension was dried, and then incubated in Sirius red solution (0.1% Sirius red in picric acid) for 1 h. After washed in 5% acetic acid, the precipitate was dissolved in 0.1 m NaOH for 30 min. The OD was measured at 550 nm and the measurements were compared to collagen standards. After cultured in OM for 3 w, alizarin red S (ARS) staining was used to assay calcium deposition or mineralization nodules on the scaffolds. The cells were fixed with 10% formalin for 10 min, then incubated with ARS stain solution (2% ARS, pH 4.2) for 30 min. After washed in distilled water three times, the ARS stained scaffolds were imaged. In addition, a calcium detection kit (Pointe Scientific) was used to quantify the calcium deposition. The calcium deposition was dissolved in 0.6 m HCl, and reacted with dye reagent. Samples were read at 570 nm wavelength, and the contents were calculated with CaCl_2_ standards. For immunofluorescence staining, the cells were fixed with 10% formalin for 15 min, permeabilized in 0.1% Triton X‐100 for 10 min and blocked in 10% BSA for 30 min. Then cells were incubated with primary antibodies at 4 °C overnight. The following primary antibodies were used for staining: goat polyclonal anti‐vWF antibodies (Santa Cruz Biotechnology) and mouse monoclonal anti‐OPN antibodies (Santa Cruz Biotechnology). After incubation with primary antibodies, donkey anti‐goat IgG‐FITC (Santa Cruz Biotechnology) and chicken anti‐mouse IgG‐TR (Santa Cruz Biotechnology) as secondary antibodies were added and incubated 1 h, respectively. Fluorescence images were observed using a confocal microscope. For immunostaining of vascular network, scaffolds were fixed in 10% formalin for 10 min, and permeabilized with Triton X‐100 (0.1%) in PBS for 10 min. After blocked with BSA for 1 h, the samples were incubated with primary antibodies (Anti‐CD31 antibody, abcam) overnight. The scaffolds were stained with chicken anti‐mouse IgG‐TR secondary antibodies (Santa Cruz Biotechnology) overnight. Finally, the hydrogels were stained with DAPI, and imaged using a confocal microscope.

*Statistical Analysis*: The data are presented as the mean ± standard deviation (SD). A one‐way analysis of variance (ANOVA) with Student's *t*‐test was used to verify statistically significant differences among groups, with *p* < 0.05 being statistically significant (^*^ *p* < 0.05; ^**^ *p* < 0.01; ^***^ *p* < 0.001).

## Supporting information

As a service to our authors and readers, this journal provides supporting information supplied by the authors. Such materials are peer reviewed and may be re‐organized for online delivery, but are not copy‐edited or typeset. Technical support issues arising from supporting information (other than missing files) should be addressed to the authors.

Supplementary

## References

[1] a) O. Tsigkou , I. Pomerantseva , J. A. Spencer , P. A. Redondo , A. R. Hart , E. O'Doherty , Y. Lin , C. C. Friedrich , L. Daheron , C. P. Lin , C. A. Sundback , J. P. Vacanti , C. Neville , Proc. Natl. Acad. Sci. USA 2010, 107, 3311; 20133604 10.1073/pnas.0905445107 PMC2840421

[2] a) W. L. Grayson , B. A. Bunnell , E. Martin , T. Frazier , B. P. Hung , J. M. Gimble , Nat. Rev. Endocrinol. 2015, 11, 140; 25560703 10.1038/nrendo.2014.234 PMC4338988

[3] T. N. Vo , F. K. Kasper , A. G. Mikos , Adv. Drug Delivery Rev. 2012, 64, 1292. 10.1016/j.addr.2012.01.016 PMC3358582 22342771

[4] O. Jeon , D. W. Wolfson , E. Alsberg , Adv. Mater. 2015, 27, 2216. 25708428 10.1002/adma.201405337 PMC4408272

[5] R. Aryal , X. P. Chen , C. Fang , Y. C. Hu , Orthop. Surg. 2014, 6, 171. 25179350 10.1111/os.12112 PMC6583294

[6] C. M. O'Brien , B. Holmes , S. Faucett , L. G. Zhang , Tissue Eng., Part B 2015, 21, 103. 10.1089/ten.teb.2014.0168 PMC4322091 25084122

[7] a) J. Wang , M. Yang , Y. Zhu , L. Wang , A. P. Tomsia , C. Mao , Adv. Mater. 2014, 26, 4961; 24711251 10.1002/adma.201400154 PMC4122615

[8] S. V. Murphy , A. Atala , Nat. Biotechnol. 2014, 32, 773. 25093879 10.1038/nbt.2958

[9] a) A. V. Do , B. Khorsand , S. M. Geary , A. K. Salem , Adv. Healthcare Mater. 2015, 4, 1742; 10.1002/adhm.201500168 PMC4597933 26097108

[10] a) P. S. Lienemann , M. P. Lutolf , M. Ehrbar , Adv. Drug Deliv. Rev. 2012, 64, 1078; 22465487 10.1016/j.addr.2012.03.010

[11] F. M. Chen , M. Zhang , Z. F. Wu , Biomaterials 2010, 31, 6279. 20493521 10.1016/j.biomaterials.2010.04.053

[12] C. J. Kearney , D. J. Mooney , Nat. Mater. 2013, 12, 1004. 24150418 10.1038/nmat3758

[13] A. C. Mitchell , P. S. Briquez , J. A. Hubbell , J. R. Cochran , Acta Biomater. 2016, 30, 1. 26555377 10.1016/j.actbio.2015.11.007 PMC6067679

[14] B. Holmes , K. Bulusu , M. Plesniak , L. Zhang , Nanotechnology 2016, 27, 064001. 26758780 10.1088/0957-4484/27/6/064001 PMC5055473

[15] S. T. Koshy , T. C. Ferrante , S. A. Lewin , D. J. Mooney , Biomaterials 2014, 35, 2477. 24345735 10.1016/j.biomaterials.2013.11.044 PMC3893146

[16] a) R. R. Costa , J. F. Mano , Chem. Soc. Rev. 2014, 43, 3453; 24549278 10.1039/c3cs60393h

[17] a) P. T. Hammond , Mater. Today 2012, 15, 196;

[18] F. Gaudiere , S. Morin‐Grognet , L. Bidault , P. Lembre , E. Pauthe , J. P. Vannier , H. Atmani , G. Ladam , B. Labat , Biomacromolecules 2014, 15, 1602. 24666097 10.1021/bm401866w

[19] a) N. J. Shah , M. N. Hyder , M. A. Quadir , N. M. Dorval Courchesne , H. J. Seeherman , M. Nevins , M. Spector , P. T. Hammond , Proc. Natl. Acad. Sci. USA 2014, 111, 12847; 25136093 10.1073/pnas.1408035111 PMC4156697

[20] N. J. Shah , M. L. Macdonald , Y. M. Beben , R. F. Padera , R. E. Samuel , P. T. Hammond , Biomaterials 2011, 32, 6183. 21645919 10.1016/j.biomaterials.2011.04.036 PMC3202614

[21] J. N. Zara , R. K. Siu , X. Zhang , J. Shen , R. Ngo , M. Lee , W. Li , M. Chiang , J. Chung , J. Kwak , B. M. Wu , K. Ting , C. Soo , Tissue Eng. Part A 2011, 17, 1389. 21247344 10.1089/ten.tea.2010.0555 PMC3079169

[22] A. H. Zisch , M. P. Lutolf , J. A. Hubbell , Cardiovasc. Pathol. 2003, 12, 295. 14630296 10.1016/s1054-8807(03)00089-9

[23] a) K. Lee , E. A. Silva , D. J. Mooney , J. R. Soc., Interface 2011, 8, 153; 20719768 10.1098/rsif.2010.0223 PMC3033020

[24] B. B. Hsu , K. S. Jamieson , S. R. Hagerman , E. Holler , J. Y. Ljubimova , P. T. Hammond , Angew. Chem. Int. Ed. Engl. 2014, 53, 8093. 24938739 10.1002/anie.201403702 PMC4387866

[25] a) J. Ma , J. J. van den Beucken , F. Yang , S. K. Both , F. Z. Cui , J. Pan , J. A. Jansen , Tissue Eng., Part C 2011, 17, 349; 10.1089/ten.TEC.2010.0215 20932081

[26] E. J. Lee , L. E. Niklason , Tissue Eng., Part C 2010, 16, 1191. 10.1089/ten.tec.2009.0652 PMC2943404 20170423

[27] K. Janeczek Portalska , A. Leferink , N. Groen , H. Fernandes , L. Moroni , C. van Blitterswijk , J. de Boer , PloS One 2012, 7, e46842. 23056481 10.1371/journal.pone.0046842 PMC3464214

[28] a) H. Cui , Y. Wang , L. Cui , P. Zhang , X. Wang , Y. Wei , X. Chen , Biomacromolecules 2014, 15, 3146; 24995801 10.1021/bm5007695

