# Supplementary material for: Biologically Inspired Smart Release System Based on 3D Bioprinted Perfused Scaffold for Vascularized Tissue Regeneration
Source: Adv Sci (Weinh). 2016 Apr 15;3(8):1600058. doi: 10.1002/advs.201600058 (PMC5074245; doi:10.1002/advs.201600058)
Supplement: Supplementary file 1 — Supplementary [file ADVS-3-0d-s001.pdf]

Copyright WILEY-VCH Verlag GmbH & Co. KGaA, 69469 Weinheim, Germany, 2016.

ADVANCED SCIENCE
Open Access

Supporting Information

for *Adv. Sci.,* DOI: 10.1002/advs.201600058

Biologically Inspired Smart Release System Based on 3D Bioprinted Perfused Scaffold for Vascularized Tissue Regeneration

*Haitao Cui, Wei Zhu, Benjamin Holmes, and Lijie Grace Zhang**

Copyright WILEY-VCH Verlag GmbH & Co. KGaA, 69469 Weinheim, Germany, 2013.

Supporting Information

Biologically Inspired Smart Release System Based on 3D Bioprinted Perfused Scaffold for Vascularized Tissue Regeneration

*Haitao Cui, Wei Zhu, Benjamin Holmes, and Lijie Grace Zhang**

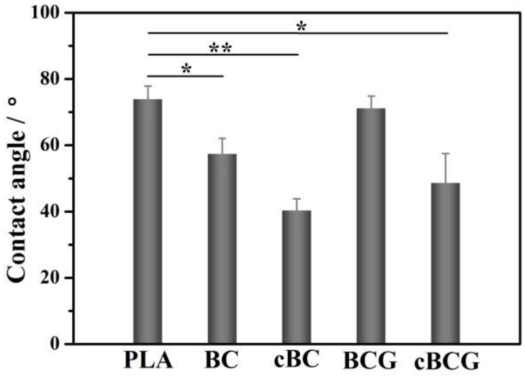

Figure S1. Contact angle measurement of different scaffold materials.

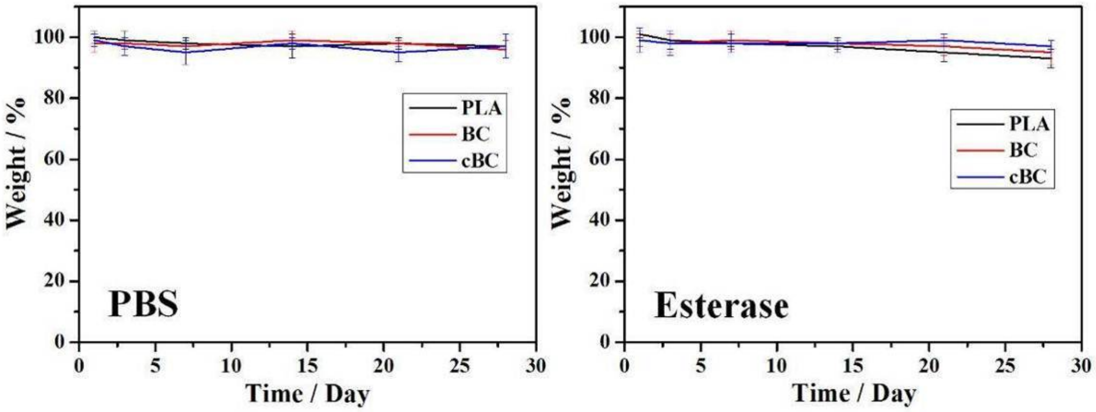

Figure S2. Degradation test of different scaffolds in PBS and esterase for 4 weeks.

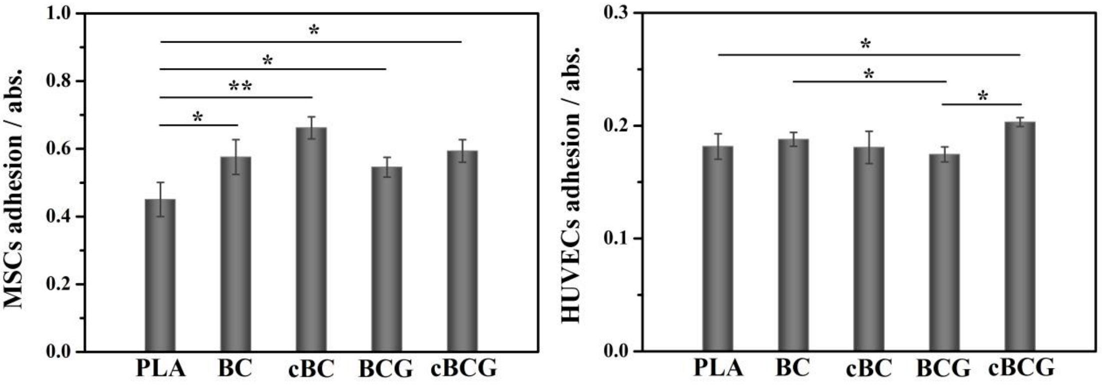

Figure S3. MSCs and HUVECs adhesion study on different scaffolds for 4 h.

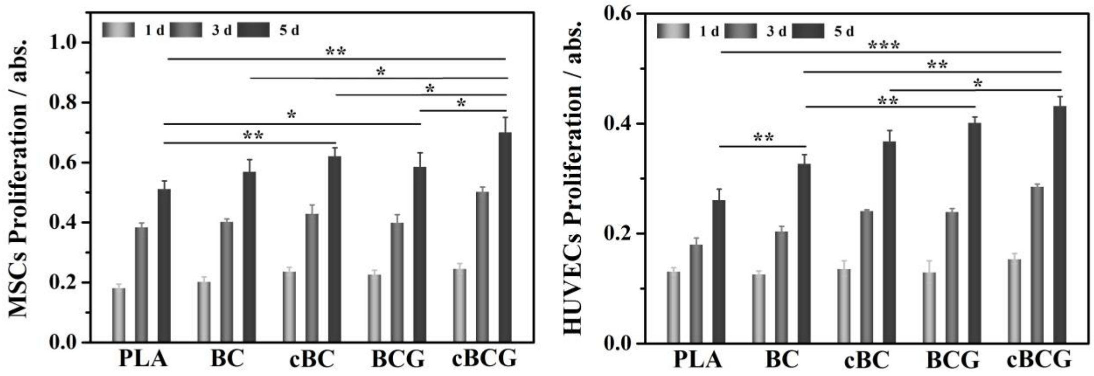

Figure S4. MSCs and HUVECs proliferation study on different scaffolds for 1, 3 and 5 days.

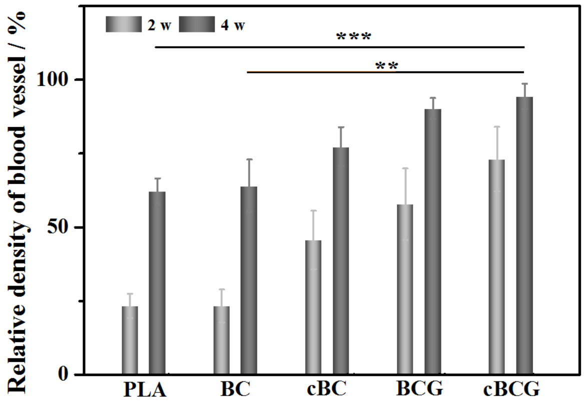

Figure S5. Relative density of blood vessel counted by CD31 staining on different scaffolds for 2 and 4 weeks.

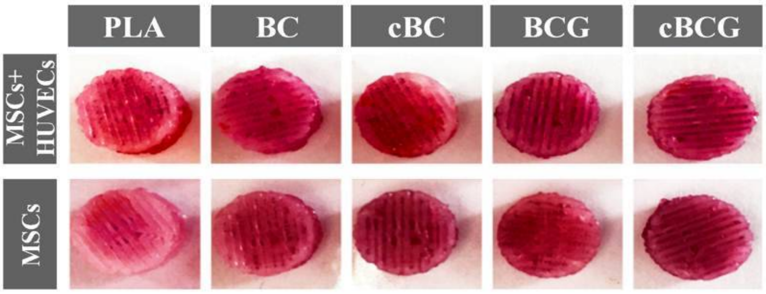

Figure S6. Mineralization of osteogenic differentiation of hMSCs and hMSCs/HUVECs on different scaffolds analyzed by Alizarin red staining.

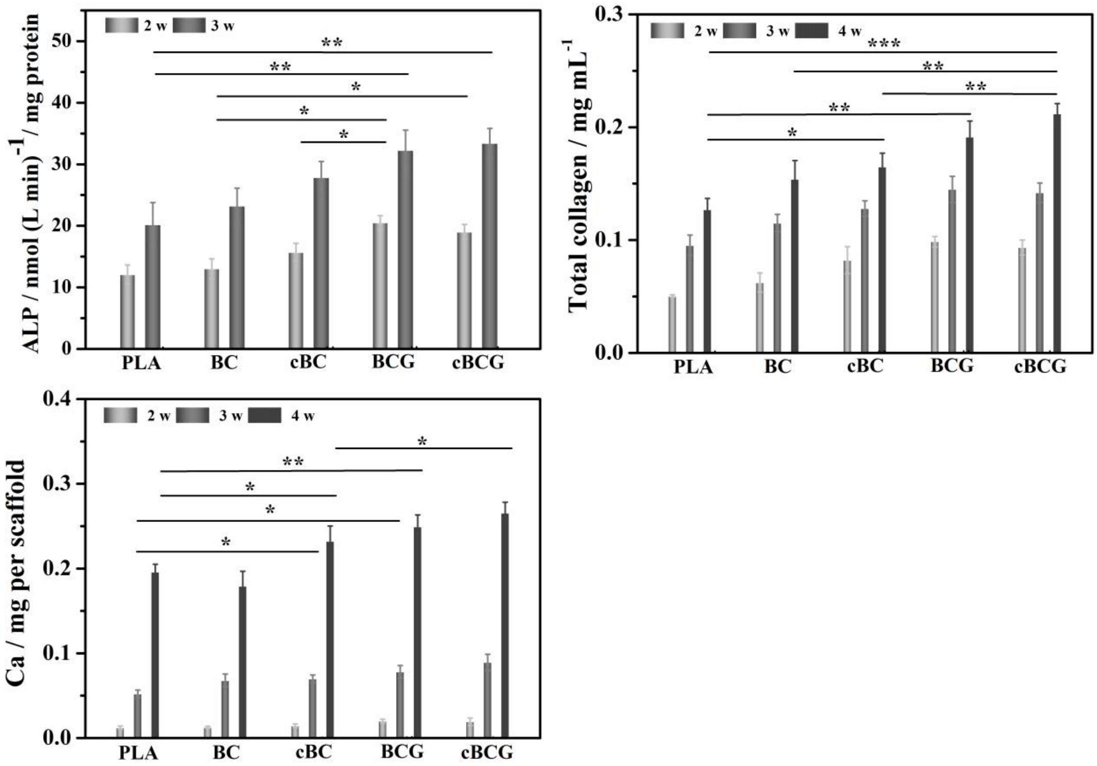

Figure S7. Osteogenic differentiation study of hMSCs/HUVECs co-culture (ALP, total collagen, calcium content) on different scaffolds in the static conditions for 4 weeks.
